# A study on the correlation between work stressors and the coping styles of outpatients and emergency nurses in 29 pediatric specialty hospitals across China

**DOI:** 10.3389/fpsyg.2022.951671

**Published:** 2022-11-04

**Authors:** Nan Song, Chun-Li Wang, Lin-Qi Zhang, Xu-Mei Wang

**Affiliations:** Department of Nursing, Beijing Children's Hospital, Capital Medical University, National Center for Children's Health, Beijing, China,

**Keywords:** pediatric hospitals, outpatient and emergency nurses, work stressors, coping style, correlation study

## Abstract

**Objective:**

This study aimed to better understand the current situation involving work stressors and the coping styles of outpatient and emergency nurses in 29 pediatric specialty hospitals across China. The study analyzed this correlation to provide a reference for the occupational stress management of pediatric nurses.

**Methods:**

From June to September 2020, 1,457 outpatient and emergency nurses in 29 pediatric specialty hospitals across China were selected as study participants, and a questionnaire survey was conducted using the Basic Information Questionnaire, the Chinese version of the Work Stressor Scale for Nurses, and the Simple Coping Style Scale.

**Results:**

The assessed stress level of outpatient and emergency nurses in 29 tertiary pediatric specialty hospitals nationwide is lower than the results of the survey of the 2007 domestic norm, *p* < 0.05. The stressors related to nurses’ expectations, family conflicts, the nature of nursing work, patient factors, and workload were lower compared with the national norm (*p* < 0.05). The positive coping style score on the Simple Coping Style Scale for pediatric outpatient nurses was (36.66 ± 6.16), and work stressors were positively associated with negative coping styles (*p* < 0.01). Multiple regression analysis showed that the influencing factors of work stressors among pediatric outpatient and emergency nurses correlated with the authorized size, age, working years of nurses, work department, and negative coping styles.

**Conclusion:**

Negative coping styles were present among pediatric outpatient and emergency nurses and were associated with work stressors. The influencing factors of stressors mainly correlated with the clinical establishment, age, years of employment as a nurse, work department, and negative coping styles.

## Introduction

Work stress, also referred to as “job stress,” “occupational stress,” or “occupational strain,” refers to the stress and strain that practitioners experience as a result of their occupation or the role they perform, as well as its related factors (While and Clark, 2021). Nursing work stress is typically defined as the stress and strain experienced by nurses while performing nursing work (Li and Yu, 2017). Pediatric outpatient and emergency nurses have heavy workloads, experience high work risk, and often need to manage emergencies (Wang and Liu, 2022). As a result, pediatric nurses often function in a high-stress state and experience heavy physiological and psychological stress, which can significantly impact their professional identity and work enthusiasm (Ma and Xie, 2022).

To alleviate the Work Stressor, many things can be done, such as encouraging hospital administrators to develop empirically tested strategies to increase decision authority and increasing social support from supervisors and staff (Labrague et al., 2018). Conversely, in some studies, personal variables such as monthly income, number in the household, years of experience, and gender did not yield significant contributions to their stress experiences (Labrague et al., 2018).

Pediatric outpatient and emergency nurses face a more challenging and unique work environment with complex interpersonal relationships that can easily cause high work stress and affect their psychological stress and coping styles (Zhou et al., 2020). Studies have shown that stressors in the professional and work-related aspects of emergency nursing positively correlate with the rationalized coping styles of nurses (Li et al., 2018). In pediatric specialty hospitals, outpatient and emergency nurses experience high work intensity and stress (S. N. Li et al., 2019). This paper investigated the work stressors and coping styles of nurses in these departments in 29 tertiary pediatric specialty hospitals across China. The authors analyzed the correlation and influencing factors between work stress and the coping styles of pediatric outpatient and emergency nurses to better understand the mental health status of nursing staff.

## Participants and methods

### Participants

From June to September 2020, outpatient and emergency nurses from 29 pediatric specialty hospitals across China were selected as the study population using a convenience sampling method. The inclusion criteria were as follows: (1) registered nurses; (2) more than 1 year of work experience in pediatric outpatient and emergency departments; (3) the nurses signed an informed consent form for inclusion in the study; and (4) nurses agreed to voluntarily complete the survey. The exclusion criteria were as follows: (1) nurses who had been on maternity/sick leave or absent from work for more than 6 months and (2) non-unit nurses.

### Methods

#### Survey instrument

The questionnaire was designed to be divided into the following three parts: (1) General information part: general information about the participants, including age, gender, marital status, childbirth status, education levels, years employed as a nurse, and job title were obtained.

(2) “Work Stressor Scale for Nurses” (Zhang et al., 2007) part: “Work Stressor Scale for Nurses” (Li et al., 2018), developed by Zhang et al. (2007), was used in this research, which comprised six factors, nurses’ expectations, work–family conflicts, interpersonal relationships at work, the nature of nursing work, patient factors, workload, and a total of 61 items. Each item was scored using a Likert 5-point scale which will contain two extreme poles and a neutral option connected with intermediate answer options. The frequency of work stressors was measured, ranging from “never encountered or experienced” to “encountered or experienced almost every day” on a scale from 1 to 5, respectively, and perceptions of stress were measured and scored from 1 to 5 (“none” to “very severe”), based on psychological perception; the product of the two scores was used to represent the score of the item. The total score for nurse work stress was obtained by adding together the scores of all the items; a higher total score reflected a higher level of work stress among nurses. The Cronbach’s alpha (*α*) coefficient for each of the scale’s six factors was 0.88–0.93, and the Cronbach’s α coefficient of the total scale was 0.97.

(3) Simplified Cope Style Questionnaire part: The Simplified Cope Style Questionnaire (Xie, 1998) developed by Xie et al. was also adopted in the current study. The questionnaire comprised two dimensions and a total of 20 items, the positive or the negative coping style. Items 1–12 compromised the first dimension reflecting the positive coping styles and items 13–20 composed the second dimension suggesting the negative coping styles. In the questionnaire, 0–3 points correspond to “Never,” “Occasionally,” “Sometimes,” and “Often” respectively, and the highest total scores for the corresponding dimensions, positive and negative, were 36 and 24, respectively. The retest reliability of the scale was 0.89, the Cronbach’s α coefficient was 0.90, and the Cronbach’s α coefficients of the two dimensions were 0.89 and 0.78.

#### Survey using an online questionnaire

Questionnaires were distributed to 29 selected pediatric general and specialized hospitals across the country. After obtaining consent, the researcher sent the “QR code” for accessing the survey to the nursing department of the corresponding hospitals, which, in turn, forwarded it to their pediatric nurses. The respondents could complete the questionnaire online using smart terminals (e.g., smartphones). Completing the questionnaire was done anonymously, and detailed instructions were included on the first page of the survey to clarify its purpose and method to help ensure the comprehensiveness and validity of the recovered questionnaires. All items were set as compulsory questions; an internet protocol address was allowed to complete the survey only once to avoid duplicate responses. A total of 1,463 surveys were collected, with 1,457 valid questionnaires (questionnaire recovery rate, 99.59%).

### Statistical processing

The SPSS 18.0 statistical software program was used to conduct data entry and analysis. Enumeration data were expressed as a frequency and composition ratio, and measurement data were expressed as mean ± standard deviation. Univariate analysis of nursing work stressors was conducted using an independent sample *t*-test and one-way analysis of variance. Multiple linear regression was employed to analyze variables that were significant in the univariate analysis, and differences were considered statistically significant at *p* < 0.05.

## Results

### General information about the survey participants

The age of the participants ranged between 20 and 59 years (39.50 ± 11.69); professional employment years were 18.71 ± 11.46; and their years employed in pediatric outpatient/emergency departments were 17.02 ± 10.93; Table 1).

**Table 1:** General information about the survey participants (*n* = 1,457).

Items	Contents	Sample size/case	Composition ratio %
Gender	Female	1,432	98.28
	Male	25	1.72
Marital status	Single	422	28.96
	Married	964	66.16
	Divorced	63	4.32
	Widowed	5	0.34
	Remarried	3	0.21
Childbearing	No children	542	37.20
	One child	704	48.32
	Two children	211	14.48
Educational background	Technical secondary school	62	4.26
	Junior college	424	29.10
	Undergraduate	962	66.03
	Postgraduate or above	9	0.62
Professional title	Nurse	365	25.05
	Nurse practitioner	627	43.03
	Nurse-in-charge	401	27.52
	Associate professor of nursing	61	4.19
	Professor of nursing	3	0.21
Specialized nurses	Yes	306	21.00
	No	1,151	79.00
Employment category	Regular employees	625	42.90
	Contract nurses	701	48.11
	Non-staff nurses	131	8.99

### The findings about the six factors of the “Work Stressor Scale for Nurses” in this study (*n* = 1,457) compared to the results of the survey of the 2007 domestic norm

Compare with the results of the survey of the 2007 domestic norm, five in six items of the work stressor score illustrated the downward trend with significant differences, *p* < 0.001.

The national normative model of the Work Stressor Scale for Nurses showed a total scale score of (566.04 ± 19.56), indicating the presence of a high level of work stress among nurses nationwide between July and December 2005 (Zhang et al., 2007). The Cronbach’s alpha (*α*) coefficient for each of the scale’s six factors was 0.88–0.93, and the Cronbach’s α coefficient of the total scale was 0.97. In this study, the assessed stress level of outpatient and emergency nurses in 29 tertiary pediatric specialty hospitals nationwide is lower than the results of the survey of the 2007 domestic norm (Zhang et al., 2007), using the same Work Stressor Scale for Nurses scale (Zhang et al., 2007; Table 2).

**Table 2:** The findings about the six factors of the “Work Stressor Scale for Nurses” in this study (*n* = 1,457) compared to the results of the survey of the 2007 domestic norm.

Sequencing	Item	Score (M ± SD)	Domestic norm	*t*	*p* value
Work stressor score for nurses	Nature of work	98.52 ± 47.89	133.11 ± 59.51	−19.08	<0.001	
	Workload	96.74 ± 42.89	119.87 ± 48.27	−14.25	<0.001	
	Interpersonal relationship at work	79.93 ± 40.61	81.52 ± 38.07	−1.04	0.300	
	Nurse expectations	73.6 ± 38.45	91.37 ± 39.14	−12.21	<0.001	
	Patient factors	64.32 ± 35.59	80.06 ± 37.15	−11.69	<0.001	
	Work–family conflict	53.54 ± 30.63	60.09 ± 30.01	−5.65	<0.001	
	Stressor score	466.64 ± 218.05	566.04 ± 19.56	−12.04	<0.001

*M* ± SD, mean ± standard deviation.

The mean score of the positive and negative coping styles assessed by the Simplified Cope Style Questionnaire (Xie, 1998) was 36.66 ± 6.16 and 17.58 ± 4.5, respectively, and the retest reliability of the Simplified Cope Style Questionnaire was 0.89, the Cronbach’s α coefficient was 0.90, and the Cronbach’s α coefficients of the two dimensions were 0.89 and 0.78 (When the reliability is greater than 0.70, it is considered acceptable).

### Univariate analysis of the different general information on work stressors of pediatric outpatient and emergency nurses

Statistical differences of the nurses’ expectations were observed in marital status, employment category, professional title, years of nursing work, and age, *p* < 0.05. There were statistical differences in work–family conflict, interpersonal relationships at work, and the nature of work among nurses with different years of nursing experience and of different ages, *p* < 0.05. Statistical differences were also observed in patient factors and workload among nurses with different employment categories and in different departments, *p* < 0.05 (Table 3).

**Table 3:** Univariate analysis of the different general information on work stressors of pediatric outpatient and emergency nurses (*n* = 1,457).

Factors		Nurse expectations	Work–family conflict	Interpersonal relationship at work	Nature of work	Patient factors	Workload	Total score
Marital status	Single	69.26 ± 37.03	50.32 ± 29.34	74.41 ± 38.71	92.84 ± 47.02	62.98 ± 31.39	94.5 ± 40.85	444.31 ± 207.93
	Married	74.5 ± 38.49	54.25 ± 30.12	81.15 ± 40.55	100.14 ± 47.25	64.48 ± 36.59	97.51 ± 43.03	472.03 ± 217.55
	Divorced/separated	82.94 ± 42.35	61 ± 39.92	91.14 ± 46.62	104.23 ± 57.49	70.83 ± 40.96	97.43 ± 46.39	507.57 ± 255.86
	Widowed	101 ± 30.79	60.33 ± 52	93.33 ± 57.05	124.67 ± 66.08	60 ± 41.07	127 ± 88.39	566.33 ± 328.75
	Remarried	21.5 ± 12.02	15 ± 2.83	37 ± 19.8	49 ± 38.18	32.5 ± 24.75	43 ± 21.21	198 ± 118.79
	F	2.446	1.896	2.223	1.629	0.765	1.318	1.754
	P	0.045	0.109	0.065	0.165	0.548	0.262	0.136
Employment category	Regular employees	70.85 ± 36.8	51.8 ± 30.47	76.88 ± 39.24	96.68 ± 47.36	60.47 ± 34.12	92.65 ± 41.66	449.32 ± 210.45
	Contract nurses	77.62 ± 39.43	55.41 ± 30.54	83.9 ± 41.08	102 ± 47.91	68.84 ± 37.1	101.66 ± 43.41	489.42 ± 222.11
	Non-staff nurses	67.22 ± 41.28	53.61 ± 32.09	75.8 ± 45.05	89.39 ± 50.31	62.12 ± 33.04	93.67 ± 45.42	441.8 ± 233.09
	*F*	3.279	1.123	2.714	2.002	4.617	3.734	3.107
	*p*	0.038	0.326	0.067	0.136	0.01	0.024	0.045
Professional title	Nurse	64.9 ± 37.43	48.74 ± 29.02	73.51 ± 41.77	89.45 ± 46.96	61.24 ± 34.16	91.21 ± 42.43	429.03 ± 215.16
	Nurse practitioner	74.51 ± 39.43	55.27 ± 32.62	81.92 ± 42.15	100.34 ± 50.7	63.72 ± 35.96	98.3 ± 44.53	474.06 ± 228.03
	Nurse-in-charge	78.17 ± 37.28	55.3 ± 29.14	81.14 ± 36.32	101.45 ± 44.05	66.89 ± 34.75	99.56 ± 41.04	482.5 ± 202.73
	Associate professor of nursing	76.49 ± 37.37	50.18 ± 27.97	83.62 ± 44.09	103.98 ± 46.74	67.6 ± 41.97	93.8 ± 40.93	475.67 ± 221.64
	Professor of nursing	47.33 ± 17.62	41 ± 12.53	49 ± 14.53	69 ± 11.27	49 ± 10.82	58 ± 12.17	313.33 ± 58.39
	F	3.053	1.559	1.663	2.034	0.79	1.587	1.826
	P	0.016	0.183	0.157	0.088	0.532	0.176	0.122
Department	Emergency	79.41 ± 35.43	55.42 ± 28.57	84.84 ± 36.79	105.1 ± 43.36	72.29 ± 37.48	101.75 ± 40.87	498.81 ± 202.21
	Outpatient	70.48 ± 39.25	55.57 ± 32.17	76.28 ± 39.74	95.28 ± 47	68.36 ± 34.69	101.99 ± 42.73	467.96 ± 220.69
	Specialized outpatient clinic	72.96 ± 38.95	52.17 ± 30.65	79.77 ± 42	97.67 ± 49.48	60.25 ± 34.8	93.15 ± 43.31	455.96 ± 221.33
	F	2.064	1.002	1.59	1.654	7.085	3.531	1.916
	P	0.128	0.368	0.205	0.192	0.001	0.03	0.148
Years of nursing work	≤2 years	57.47 ± 35.55	41.15 ± 25.56	61.77 ± 33.2	76.85 ± 39.72	58.64 ± 32.37	85.32 ± 40.59	381.21 ± 190.5
	2–5 years	72.18 ± 37.67	55.11 ± 30.35	81.75 ± 40.89	98.6 ± 47.65	67.51 ± 32.63	100.36 ± 39.6	475.51 ± 212.93
	6–10 years	73.72 ± 37.7	54.21 ± 29.93	80.42 ± 40.23	97.82 ± 48.1	63.46 ± 36.33	97.02 ± 44.35	466.66 ± 219.06
	11–19 years	75.66 ± 40.94	56.23 ± 32.14	81.78 ± 43.56	100.46 ± 50.3	65.55 ± 36.91	100.19 ± 45.16	479.87 ± 231.14
	≥20 years	76.57 ± 36.4	52.56 ± 30.31	81.73 ± 38.15	103.25 ± 45.77	63.67 ± 35.81	93.86 ± 40.61	471.64 ± 207.56
	F	2.81	2.73	2.93	3.28	0.62	1.66	2.31
	P	0.03	0.03	0.02	0.01	0.65	0.16	0.06
Age	< 30	67.71 ± 35.57	50.17 ± 27.86	74.74 ± 38.04	91.54 ± 45.24	60.96 ± 31.08	93.86 ± 40.92	438.98 ± 200.97
	31–40	76.51 ± 41.65	56.87 ± 33.02	83.18 ± 43.95	101.05 ± 50.65	67.4 ± 39	100.31 ± 46.05	485.32 ± 237.62
	41–50	80.48 ± 38.27	56.1 ± 32.83	85.68 ± 40.81	107.69 ± 47.02	64.43 ± 38.04	98.11 ± 40.86	492.48 ± 217.19
	> 50	71.43 ± 30.57	46.26 ± 21.04	74.83 ± 29.34	98.46 ± 43.93	65.04 ± 31.01	88.46 ± 38.89	444.48 ± 176.58
	F	3.96	3.3	3.09	3.65	1.45	1.63	2.81
	P	0.01	0.02	0.03	0.01	0.23	0.18	0.04

### Comparison of the correlation between work stress and the coping styles of pediatric outpatient and emergency nurses

The stress levels of pediatric outpatient nurses negatively correlated with positive coping styles and positively correlated with negative coping styles (*p* < 0.01; Table 4).

**Table 4:** Correlation between work stressors and coping styles of pediatric outpatient and emergency nurses (*n* = 1,457).

	Nurse expectations	Work–family conflict	Interpersonal relationship at work	Nature of work	Patient factor	Workload	Total dimension of stressors
Positive coping style	−0.183^**^	−0.174^**^	−0.179^**^	−0.125^**^	−0.113^**^	−0.059	−0.148^**^
Negative coping styles	0.267^**^	0.279^**^	0.269^**^	0.294^**^	0.253^**^	0.256^**^	0.292^**^

** *p* < 0.01.

### Multiple regression analysis of the work stressors among pediatric outpatient and emergency nurses

Multiple linear regression analysis was conducted using the six factors of the Work Stressor Scale for Nurses as the dependent variables and the statistically significant factors in the univariate analysis as the independent variables, with marriage status, clinical establishment, title, and department set as sub-variables. The results indicated establishment and negative coping styles as influencing factors of workload; age, coping style, and years of nursing experience were influencing factors regarding the nature of nurses’ work; establishment, age, and coping styles were influencing factors of nurses’ expectations; age and coping styles were influencing factors of interpersonal relationships at work; age, coping styles, and years working as a nurse were influencing factors of work–family conflicts; establishment, work department, and coping styles were influencing factors of patient factors (Table 5).

**Table 5:** Multiple regression results of stressors for workload, nature of work, nurses’ expectations, interpersonal relationships at work, work–family conflict, and patient factors.

Factors	*R* ^2^	Variable	Unstandardized coefficients		Standardized coefficients	*t*	Sig.	95% CI
			*B*	Std. error	Beta			
Workload	8.8		43.002	7.220		5.956	<0.001	28.823, 57.181
		Contract nurses	12.861	3.578	0.147	3.594	<0.001	5.834, 19.887
		Non-staff nurses	2.095	6.439	0.013	0.325	0.745	−10.550, 14.739
		Negative coping styles	2.580	0.375	0.268	6.890	<0.001	1.845, 3.316
Nature of work	12.6		61.326	19.483		3.148	0.002	23.063, 99.590
		31–40 years old	18.293	6.856	0.184	2.668	0.008	4.829, 31.757
		41–50 years old	35.564	14.202	0.291	2.504	0.013	7.672, 63.455
		>50 years old	26.892	15.541	0.144	1.730	0.084	−3.630, 57.413
		11–19 years	11.752	12.404	0.111	0.947	0.344	−12.610, 36.113
		6–10 years	19.647	13.382	0.171	1.468	0.143	−6.635, 45.928
		2–5 years	31.826	14.834	0.220	2.146	0.032	2.694, 60.958
		≤2 years	12.903	15.490	0.070	0.833	0.405	−17.519, 43.324
		Positive coping style	−1.352	0.307	−0.169	−4.407	<0.001	−1.955, −0.750
		Negative coping style	3.304	0.413	0.308	8.006	<0.001	2.493, 4.114
Nurse expectations	16.9		48.834	16.556		2.959	0.003	16.318, 81.349
		Contract nurses	19.691	4.033	0.249	4.883	<0.001	11.770, 27.611
		Non-staff nurses	12.107	6.504	0.084	1.861	0.063	−0.668, 24.881
		31–40 years old	12.953	5.613	0.162	2.308	0.021	1.929, 23.978
		41–50 years old	28.503	11.582	0.289	2.461	0.014	5.757, 51.250
		>50 years old	19.225	13.029	0.128	1.476	0.141	−6.364, 44.813
		Positive coping style	−1.385	0.243	−0.214	−5.699	<0.001	−1.862, −0.908
		Negative coping style	2.542	0.327	0.293	7.768	<0.001	1.900, 3.185
Interpersonal relationship at work	14.1		85.266	17.675		4.824	<0.001	50.553, 119.979
		31–40 years old	16.640	5.747	0.199	2.896	0.004	5.354, 27.926
		41–50 years old	22.246	11.919	0.215	1.866	0.062	−1.163, 45.655
		>50 years old	8.684	13.161	0.055	0.660	0.510	−17.162, 34.530
		Positive coping style	−1.481	0.257	−0.219	−5.762	<0.001	−1.985, −0.976
		Negative coping style	2.642	0.346	0.291	7.646	<0.001	1.964, 3.321
Work–family conflict	14.1		40.733	12.435		3.276	0.001	16.312, 65.155
		31–40 years old	12.852	4.376	0.201	2.937	0.003	4.259, 21.446
		41–50 years old	18.557	9.064	0.236	2.047	0.041	0.755, 36.359
		>50 years old	8.364	9.919	0.070	0.843	0.399	−11.116, 27.845
		11–19 years	7.160	7.917	0.105	0.904	0.366	−8.389, 22.708
		6–10 years	12.113	8.541	0.164	1.418	0.157	−4.661, 28.887
		2–5 years	21.236	9.468	0.228	2.243	0.025	2.643, 39.830
		≤2 years	8.997	9.886	0.076	0.910	0.363	−10.419, 28.413
		Positive coping style	−1.128	0.196	−0.219	−5.761	<0.001	−1.513, −0.744
		Negative coping style	2.117	0.263	0.306	8.039	<0.001	1.600, 2.635
Patient factors	11.2		46.088	10.096		4.565	<0.001	26.260, 65.916
		Contract nurses	10.893	2.964	0.149	3.676	<0.001	5.073, 16.714
		Non-staff nurses	1.974	5.323	0.015	0.371	0.711	−8.480, 12.427
		Emergency Department	6.071	3.513	0.070	1.728	0.084	−0.827, 12.970
		Outpatient Department	9.140	3.666	0.099	2.493	0.013	1.940, 16.340
		Positive coping style	−0.781	0.232	−0.130	−3.373	0.001	−1.236, −0.326
		Negative coping style	2.243	0.311	0.279	7.220	<0.001	1.633, 2.853

## Discussion

This study discovered that the nature of nursing work, workload, coworker interactions, nurses’ expectations, patient factors, and work–family conflict were the top work stressors for pediatric outpatient and emergency nurses, in that order of importance. According to the study’s findings, pediatric outpatient and emergency nurses’ workloads were statistically different from their clinical settings and their negative coping behaviors. The nature of the nursing job, age, and years of experience, as well as the nature of nursing work and the unfavorable coping mechanisms of pediatric outpatient and emergency nurses, were all statistically different among work stresses, according to this study. This study discovered a statistically significant relationship between coping strategies and patient factors among pediatric outpatient and emergency nurses, as well as between patient factors and clinical setting, work department, and work stressors. This study discovered a statistical difference between pediatric outpatient and emergency nurses’ predictions regarding work stressors, age, and clinical setting, as well as between their expectations regarding work stressors and harmful coping mechanisms. These findings were in line with prior research on workload, nursing work characteristics, nurses’ expectations, and occupational burnout(Ilić et al., 2017). The “three-shift” work schedule of pediatric outpatient and emergency nurses, as well as chronic work overload, a stressful atmosphere, and these results could all be contributing factors.(D'Ettorre et al., 2020) This revealed that as their workload rose, contract nurses were more prone than nurses who are permanently employed by a clinical establishment to feel stressed and tense. Additionally, the majority of contract nurses exhibited a poor coping mechanism. Contract nurses can be directed and motivated in their professional duties and their workforce can be stabilized by using a performance management system (Kuo et al., 2020). This revealed that more experienced elder nurses had to deal with higher levels of stress brought on by their line of work. Given their expert titles and wealth of nursing expertise, nurses with 11 to 19 years of experience should receive special attention. These nurses frequently serve as the backbone of clinical departments, carry out more teaching and research activities, and face more pressures than younger nurses (Li, L et al., 2019). This indicated that compared to nurses who were engaged permanently, contract nurses expected higher recognition, equivalent compensation for equal effort, and promotion prospects. The longer nurses tended to use negative coping mechanisms like avoidance and inattention, the more they expected breakthroughs and successes at work and the older they were. As a result, the buildup of various unpleasant emotions will probably have an impact on their psychological health (Ezenwaji et al., 2019). This shows that interpersonal ties between older (>50 years) nurses and a stressful department environment may have a direct impact on older nurses’ self-approval and worse job satisfaction (Vahedian-Azimi et al., 2019). It is hypothesized that older nurses with more work experience have dealt with more work and family conflict. Nursing managers ought to focus more on nurses between the ages of 31 and 40 who must simultaneously work, care for their families, and educate their children. They can deal with difficulties at work and the home more effectively by developing a healthy coping mechanism (Sun et al., 2018). This was particularly highlighted by the following: (1) The demanding workload of pediatric nurses, the poor collaboration and poor communication of most young children, and the complexity of nursing operations, all of which necessitate highly skilled operational abilities from nurses. (2) Nurses must have great professional knowledge and prescient talents because the conditions of children in emergency and outpatient clinics can change quickly. In addition, some illnesses are prone to return and may not be easily recognized. (3) Pediatric outpatient and emergency nurses work a lot of shifts and have erratic schedules that leave them with little time for their families and kids. They are frequently mentally and physically exhausted, which causes excessive physiological and psychological stress that can easily result in anxiety and job burnout. After the coronavirus-2019 (COVID-19) outbreak, the current study’s questionnaire was created. Emergency and outpatient nurses were subjected to a higher risk of viral infection during this time. They had to follow tight cleaning and isolation procedures, be up-to-date on pandemic prevention and control, and be proficient at donning and taking off protective clothes. They were also constantly involved in the care of high-risk or suspected COVID-19-positive children. As a result, pediatric outpatient and emergency nurses have long been exposed to high levels of stress.

To safeguard their physical and mental health, improve the caliber of their work, and boost productivity, pediatric outpatient and emergency nurses must deal with the high levels of stress they encounter. The rights and interests of nurses should also be protected, and their attitudes about their professions should be modified, by increasing nurse salaries and staffing numbers. Policies for promotions and opportunities for further education should be implemented to promote employees’ interest in their work. When adopting emergency team building in the present pandemic scenario (and in general pandemic/epidemic prevention and control), it is important to consider the emotional and psychological health of nurses.

To develop their operational and communication skills, nurses should be able to actively participate in reading sessions, emergency drills, role-playing, intensive operational training, situational simulation training, and reading sessions. They should also work on developing a fearless attitude and the ability to calmly handle a variety of situations. The Guidance on Promoting the Reform and Development of the Nursing Service Industry (Yang, 2019) makes it clear that nursing professionals and technicians should be given more time and energy to deepen their specialties and perform clinical work. Training specialist nurses serve as an important assurance for stimulating the vitality of a high-quality nursing team. To fully support the role of essential skills, the hospital offers clinical specialty guidance, facilitates discussions on difficult and critical cases, and conducts nursing specialty research. By opening nursing specialty consultation clinics and facilitating nursing workshops, the hospital strengthens nurses’ professional values and identities and increases the influence of the nursing profession (State Health Commission of the people's Republic of China, 2018). The hospital encourages nurses to re-learn and re-train, transform passive learning into active learning, and deal with the pressures and challenges brought on by the demanding standards of the nursing profession using a positive mindset and methods by carrying out individual nursing sessions, clinical teaching lectures, nursing operations, and other competitive activities.

The hospital will be able to enhance the effectiveness and quality of nursing management by adopting “magnetic hospital management principles,” creating a high-quality nursing practice environment, encouraging innovation and creativity among nurses, and fostering team unity (Wu et al., 2019; Vallone et al., 2020).

In addition, high-quality professional nurses can be drawn to the organization and help define professional values while lowering work-related weariness, increasing nurse retention, and boosting nurses’ job satisfaction, all of which help patients have a better overall prognosis (Rodríguez-García et al., 2020). Along with the management of positions, vertical nursing management should be implemented.(Zhou et al., 2016) Job descriptions must be created, and nursing performance coefficients and incentive protocols must be established based on job coefficients, risk intensity, frequency of night shifts, day-shift posts, and management posts.(Efendi et al., 2019) Compound leverage coefficient management for night shift performance, subsidies, a pay system that rewards greater productivity, and respect for the worth of nurses’ labor should all be introduced. Nurses’ motivation should also be improved (Zhou et al., 2016; Efendi et al., 2019; Rodríguez-García et al., 2020; Vallone et al., 2020). It is important to pay attention to nurses’ working hours (such as overtime, meetings, and inconspicuous time spent learning and training on the job), build response protocols, improve nurses’ work rosters, adopt flexible scheduling, and plan vacations in accordance with workload.

Through the development of a nursing brand team and the encouragement of the establishment of specialized nursing units, such as “warm-hearted service,” “advanced technology,” “superior health education,” “lean management,” and “innovation,” nurses should be encouraged to be devoted to their department, participate in management, and share honor and potential discomfort. Innovative nursing unit slogans can be used to uphold a unified, pleasant, and upbeat environment, create harmonious interpersonal interactions, and create a unified nursing culture and vision.(Ezenwaji et al., 2019; L. Li et al., 2019; Vahedian-Azimi et al., 2019) Along with stress reduction intervention management techniques like relaxation training (Catherine Calder Calisi, 2017), a mindfulness station, psychological counseling, positive meditation (van der Riet et al., 2018), and calming yoga, “dynamic management mechanisms,” such as group-building activities, parent–child activities, family visiting days, and learning and sharing sessions should be implemented to enhance nurses’ awareness and assessment of stress factors and to aid them in better managing patients.

Nursing managers help design nurses’ professional development trajectories and determine career development directions. This is accomplished by, among other things, imparting competence and pertinent abilities, managing, and carrying out scientific research based on the traits and skills of each nurse, while comparable training can be offered to help them recognize their value (Bardhan et al., 2019; Wan, 2019). To reduce the stress and negative emotional buildup experienced by nurses at work, managers should establish a role adjustment mechanism that can rotate or transfer senior nurses, nurses who may be in poor physical condition, and nurses who may exhibit incompetence in outpatient or emergency work on a regular or irregular basis (Lee and Jang, 2020). This will also ensure reasonable and fair rules. By enhancing their care and raising the regularization quota, contract nurses might be encouraged to work harder and develop a sense of “ownership” through the implementation of a long-term incentive system. In addition, lectures on traditional virtues should be given, advanced model selection should be put into place, and nurse–patient thanksgiving exchanges and other values can be put into action to improve nurses’ professional expectations and values. The staffing ratio of permanently employed nurses and contract nurses should also be adjusted (Bardhan et al., 2019; Wan, 2019; Lee and Jang, 2020).

The most significant advantage of this work is that it was conducted in 29 specialized pediatric hospitals with an adequate sample size and a large workload and made a comparison with the results of the survey of the 2007 domestic norm to find out the changes over time. This study also has some limitations as follows: Firstly, the research period of the current study coincided with the COVID-19 outbreak, and the goal of combatting the pandemic together inspired nurses’ sense of professional duty and cooperation among nurses, which may lead to a more harmonious and supportive interpersonal relationships and cause the results bias. Secondly, this study took an online questionnaire to collect results, the interpretation of the questionnaire may be insufficient, and there may be biases caused by personal subjective understanding errors.

## Summary

In summary, the stress level of outpatient and emergency nurses in 29 tertiary pediatric specialty hospitals nationwide is lower than the results of the survey of the 2007 domestic norm but remains high. This may indicate that the stressors of pediatric nurses in China had been alleviated and controlled. To alleviate work stress, nurses can be motivated by a sense of professional honor, and the support and understanding of their family members. Meanwhile, from the perspective of organizational interventions, increasing social support, providing psychological and spiritual support, and supplying with stress management interventions can be meaningful.

## Data availability statement

The original contributions presented in the study are included in the article/supplementary material, further inquiries can be directed to the corresponding author.

## Ethics statement

The studies involving human participants were reviewed and approved by the ethics committee of Beijing Children’s Hospital. The patients/participants provided their written informed consent to participate in this study.

## Author contributions

NS and X-MW: conception and design of the research and statistical analysis. NS: acquisition of data, obtaining financing, and writing of the manuscript. C-LW and L-QZ: analysis and interpretation of the data. C-LW and X-MW: critical revision of the manuscript for intellectual content. All authors contributed to the article and approved the submitted version.

## Funding

This work was supported by the Nursing Fund of Beijing Children’s Hospital, Capital Medical University (No. YHL202010), and Nursing Research Project of Chinese Medical Association Magazine (No. CMAPH-NRI2021037).

## Conflict of interest

The authors declare that the research was conducted in the absence of any commercial or financial relationships that could be construed as a potential conflict of interest.

## Publisher’s note

All claims expressed in this article are solely those of the authors and do not necessarily represent those of their affiliated organizations, or those of the publisher, the editors and the reviewers. Any product that may be evaluated in this article, or claim that may be made by its manufacturer, is not guaranteed or endorsed by the publisher.

## References

[ref1] Bardhan R. Heaton K. Davis M. Chen P. Dickinson D. A. Lungu C. T. (2019). A cross sectional study evaluating psychosocial job stress and health risk in emergency department nurses. Int. J. Environ. Res. Public Health 16:3243. doi: 10.3390/ijerph16183243, PMID: 31487874 PMC6765813

[ref2] Calisi C. C. (2017). The effects of the relaxation response on nurses' level of anxiety, depression, well-being, work-related stress, and confidence to teach patients. J. Holist. Nurs. 35, 318–327. doi: 10.1177/0898010117719207, PMID: 28720029

[ref3] D'Ettorre G. Pellicani V. Caroli A. Greco M. (2020). Shift work sleep disorder and job stress in shift nurses: implications for preventive interventions. Med. Lav. 111, 195–202. doi: 10.23749/mdl.v111i3.9197, PMID: 32624561 PMC7809943

[ref4] Efendi F. Kurniati A. Bushy A. Gunawan J. (2019). Concept analysis of nurse retention. Nurs. Health Sci. 21, 422–427. doi: 10.1111/nhs.12629, PMID: 31270927

[ref5] Ezenwaji I. O. Eseadi C. Okide C. C. Nwosu N. C. Ugwoke S. C. Ololo K. O.. (2019). Work-related stress, burnout, and related sociodemographic factors among nurses: implications for administrators, research, and policy. Medicine 98:e13889. doi: 10.1097/MD.0000000000013889, PMID: 30653094 PMC6370177

[ref6] Ilić I. M. Arandjelović M. Ž. Jovanović J. M. Nešić M. M. (2017). Relationships of work-related psychosocial risks, stress, individual factors and burnout – questionnaire survey among emergency physicians and nurses. Med. Pr. 68, 167–178. doi: 10.13075/mp.5893.00516, PMID: 28345677

[ref7] Kuo F.-L. Yang P.-H. Hsu H.-T. Su C.-Y. Chen C.-H. Yeh I.-J.. (2020). Survey on perceived work stress and its influencing factors among hospital staff during the COVID-19 pandemic in Taiwan. Kaohsiung J. Med. Sci. 36, 944–952. doi: 10.1002/kjm2.12294, PMID: 32815248 PMC7461417

[ref8] Labrague L. J. McEnroe-Petitte D. M. Leocadio M. C. Peter V. B. Cummings G. G. (2018). Stress and ways of coping among nurse managers: an integrative review. J. Clin. Nurs. 27, 1346–1359. doi: 10.1111/jocn.14165, PMID: 29148110

[ref9] Lee E. Jang I. (2020). Nurses’ fatigue, job stress, organizational culture, and turnover intention: a culture-work-health model. West. J. Nurs. Res. 42, 108–116. doi: 10.1177/0193945919839189, PMID: 31007153

[ref10] Li L. Ai H. Gao L. Zhou H. Liu X. Zhang Z.. (2019). Moderating effects of coping on work stress and job performance for nurses in tertiary hospitals: a cross-sectional survey in China. J. Nurs. Scholarsh. 53, 217–226. doi: 10.1186/s12913-017-2348-3 PMC5469137 28606180

[ref11] Li S. N. Cai R. Y. Cai W. J. Wang Q. (2019). Correlation between job stress, coping style and social support of neonatal nurses in Hainan province. Guangdong Med. J. 40, 289–292. doi: 10.13820/j.cnki.gdyx.20183769

[ref12] Li C. X. Fang Y. W. Yuan S. Y. Wang G. Y. Ding Y. Z. Tian S. Z. (2018). Correlation analysis between stress and coping styles of emergency room nurses in class III grade a hospitals. Mod. Nurs. 24, 1184–1186. doi: 10.3760/cma.j.issn.1674-2907.2018.10.013

[ref13] Li J. Yu H. Y. (2017). Mediating effect of coping styles between job stressors and job burnout among contract nurses. Ind. Health Occup. Dis. 43, 208–212.

[ref15] Ma X. F. Xie M. (2022). Current situation of work stress of pediatric nurses and potetial influencing factors. Int. J. Nurs. 41, 2157–2161. doi: 10.3760/cma.j.cn221370-20201201-00556

[ref16] Rodríguez-García M. C. Márquez-Hernández V. V. Belmonte-García T. Gutiérrez-Puertas L. Granados-Gámez G. (2020). Original research: how magnet hospital status affects nurses, patients, and organizations: a systematic review. Am. J. Nurs. 120, 28–38. doi: 10.1097/01.NAJ.0000681648.48249.16, PMID: 32541337

[ref17] State Health Commission of the people's Republic of China (2018). Guidance on promoting the reform and development of nursing service industry. Chin. J. Pract. Rural Doctors 25:4.

[ref18] Sun J. Lian D. M. Cheng L. Bo H. X. Lu P. W. (2018). Survey of job burnout and coping styles of pediatric nurses in 5 third grade hospitals in Beijing. Chin. Nurs. Res. 32, 2541–2544. doi: 10.12102/j.issn.1009-6493.2018.16.014

[ref19] Vahedian-Azimi A. Hajiesmaeili M. Kangasniemi M. Fornés-Vives J. Hunsucker R. L. Rahimibashar F.. (2019). Effects of stress on critical care nurses: a National Cross-Sectional Study. J. Intensive Care Med. 34, 311–322. doi: 10.1177/0885066617696853, PMID: 29277137

[ref20] Vallone F. Smith A. P. Zurlo M. C. (2020). Work-related stress and wellbeing among nurses: testing a multi-dimensional model. Jpn J. Nurs. Sci. 17:e12360. doi: 10.1111/jjns.12360 32803915

[ref21] van der Riet P. Levett-Jones T. Aquino-Russell C. (2018). The effectiveness of mindfulness meditation for nurses and nursing students: an integrated literature review. Nurse Educ. Today 65, 201–211. doi: 10.1016/j.nedt.2018.03.018, PMID: 29602138

[ref22] Wan Y. (2019). Influence of occupational expectation and occupational values of emergency nurses on their prospective coping styles. Occup. Health 35, 3095–3100.

[ref23] Wang Y. Liu Y. P. (2022). Correlation between work stress, coping style and mental health status of nurses in pediatric intensive care unit. Int. J. Nurs. 41, 2351–2354. doi: 10.3760/cma.j.cn221370-20200816-00604

[ref24] While A. E. Clark L. L. (2021). Management of work stress and burnout among community nurses arising from the COVID-19 pandemic. Br. J. Commun. Nurs. 26, 384–389. doi: 10.12968/bjcn.2021.26.8.384, PMID: 34343046

[ref25] Wu X. J. Zhu C. Jiao J. (2019). Magnet hospital concept: to create a high-quality nursing practice environment. J. Nurs. Adm. 19, 305–308.

[ref26] Xie Y. N. (1998). A preliminary study on the reliability and validity of simple coping style scale. Chin. J. Clin. Psych. 2, 114–115.

[ref14] Yang M. Y. (2019). Analysis of job stressors and related factors of emergency nurses. J. Practical Clin. Nurs. 4:134.

[ref27] Zhang J. P. Yao S. J. Tang Y. (2007). The development and psychometric analysis of nurse stressor scale. Chin. J. Nurs. 42, 396–401.

[ref28] Zhou W. J. Gu S. Shang S. M. (2016). A qualitative study on application of vertical management in nursing position management. J. Nurs. Sci. 31, 60–63.

[ref29] Zhou Z. Hu F. Gu Y. (2020). Study on the correlation between occupational stressors and mental load in pediatric emergency nurses. Shanghai Nurs. 20, 36–38.

